# Learning curve of ultrasound-guided caudal epidural block: a CUSUM pivotal analysis

**DOI:** 10.3389/fmed.2025.1624205

**Published:** 2025-09-08

**Authors:** Dongmei Ma, Ping Chen, Jianhong Xu

**Affiliations:** Department of Anesthesiology, The Fourth Affiliated Hospital of School of Medicine, International School of Medicine, International Institutes of Medicine, Zhejiang University, Yiwu, China

**Keywords:** learning curve, ultrasound-guided, caudal epidural block, CUSUM analysis, teaching

## Abstract

**Background and objectives:**

This study aims to dynamically evaluate the learning curve of anesthesiology residents’ mastery of ultrasound-guided caudal epidural block (US-CEB) through cumulative summation (CUSUM) analysis, providing a quantitative basis for optimizing the training program.

**Methods:**

After ethical approval and registration, 10 novice anesthesiology residents underwent standardized training in US-CEB. Over 4 months, each of 10 residents performed 30 procedures, totalling 300 cases on 300 patients undergoing perineal and sacrococcygeal surgeries. The CUSUM analysis was applied to measure performance in terms of success rates, procedural times and self-confidence score.

**Results:**

The learning curve had two phases: rapid skill acquisition followed by a plateau indicating proficiency. The median time for participants to identify landmarks was 49.5 s, and the US-CEB procedure took 146.6 s. Landmark identification skills plateaued after about 9 procedures, US-CEB skills after 11, and self-confidence after 13. Polynomial modeling showed a strong non-linear relationship between procedures and performance, with high *R*^2^ values.

**Conclusion:**

The study shows that US-CEB can be learned quickly with targeted training. A structured initial training and deliberate practice help residents master ultrasound-guided sacral canal block procedures. As operators improve their skills, their confidence increases, fostering continuous development and mastery.

## Introduction

The pursuit of medical expertise constitutes a lifelong endeavor, with residency training serving as a pivotal component of this educational continuum (1). In the field of anesthesiology, residents are required to master a diverse array of clinical skills healthcare (2, 3). Ultrasound-guided regional anesthesia has revolutionized regional anesthesia by offering real-time imaging that enhances the precision and safety of procedures, significantly improving anesthesia safety and transforming nerve block education. With ultrasound guidance, the anatomy, puncture techniques, and skills are now more accessible and comprehensible to students compared to the traditional blind puncture methods. This has not only facilitated a more straightforward learning process for students but also increased practice efficiency and patient safety.

Mastery of clinical skills, especially within the realm of anesthesia, has historically been conceptualized through the “learning curve” framework (4). In the field of anesthesiology, the importance of the learning curve is particularly evident in mastering procedural skills like tracheal intubation (5) and epidural anesthesia (6). While learning curves for ultrasound-guided peripheral nerve blocks have been extensively documented (7), the specific learning pathway for ultrasound-guided caudal epidural block (US-CEB), which involves distinct anatomical challenges such as identifying the sacral hiatus and navigating the sacrococcygeal ligament, remains poorly characterized. This journey toward competency is iterative, necessitating a cycle of practice, feedback, and refinement. The specific learning curve for US-CEB remains insufficiently studied, and a clearer understanding is essential for developing effective training modules aimed at enhancing patient safety and improving anesthesiologists’ skill levels.

Research has shown that ultrasound imaging can significantly enhance the success rate of caudal blocks, especially in patients with difficult surface anatomic landmarks (8). The ability to visualize the anatomy in real-time allows practitioners to make more informed decisions regarding needle placement, thereby reducing the number of attempts required for successful dural puncture (8). In addition to improving success rates, ultrasound guidance also aids in understanding the dynamics of local anesthetic spread within the epidural space. Observational studies have documented patterns of secondary spread and redistribution of local anesthetics, which are critical for optimizing the effectiveness of the block (9).

Despite the advantages of US-CEB, the transition to this technique requires a significant learning curve, demanding the acquisition of both technical and interpretative skills. Residents must not only perform the block with technical proficiency but also accurately interpret the ultrasound images, a process that is complex and requires extensive practice and feedback.

The Cumulative Summation Analysis (CUSUM) has emerged as a valuable tool in medical education for evaluating the learning curves of various procedures (10). It provides a graphical representation of progress over time by plotting the cumulative sum of performance deviations from a target level, offers a visual representation of an individual’s progress toward a defined level of competence (10).

The objective of this study is to utilize CUSUM analysis to assess the learning curve of US-CEB, with a specific focus on landmarks identification, US-CEB procedural times, and self-confidence of residents. Through this analysis, this study aims to make a valuable contribution to training programs that enhance patient safety and improve the skill level of anesthesiologists performing US-CEB procedures.

## Methods

This study was approved by the Ethics Committee of the Fourth Affiliated Hospital of Zhejiang University Medical College (Yi Wu, People’s Republic of China) (NO: KY-2024-030). The trial was registered with the Clinical Trial Registry in the 03/07/2024 (No: NCT06290752). The period of enrollment was from March 2024 to July 2024, 30 consecutive cases were selected for each of 10 anesthesiology residents, totaling 300 cases. All residents had comparable foundational training in ultrasound but were novices in terms of hands-on experience with US-CEB and CEB, ensuring a level starting point in their technical knowledge. This study received approval from the institutional review board, and written informed consent was obtained from all participants to ensure adherence to ethical standards and respect for participant autonomy.

A total of 300 patients scheduled for elective anal fistulectomy or hemorrhoidectomy were chosen based on specific criteria: age 18–65, ASA physical status I to II, and BMI 18–35 kg/m2. Exclusion criteria included local infection, hypersensitivity to local anesthetics, and preexisting neuralgic or spinal disease.

### Training and study

Upon being admitted to the anesthesiology department, the 10 novice anesthesiology residents were introduced to a comprehensive training program designed to equip them with the essential skills for US-CEB procedures. The training began with technical guidance provided by an experienced attending physician special in the field. This initial phase aimed to acquaint the residents with the theoretical aspects and practical nuances of the procedure.

Following, the residents were presented with a detailed operation video that demonstrated the US-CEB procedure in its entirety. This visual aid effectively facilitated the residents to observe the intricate steps involved in the process, from patient preparation to landmarks Identification and final puncture by ultrasound. The video was thoughtfully curated to accentuate the critical elements that are often challenging for novices to grasp.

In addition to the video, the residents were also provided with the opportunity to observe a live clinical operation demonstration conducted by the same experienced instructor. This hands-on observation was crucial for the residents to correlate the theoretical knowledge and video observations with real-life clinical scenarios. The live demonstration enabled the residents to observe the instructor’s techniques, decision-making processes, and how they adapted to unforeseen challenges that may arise during the procedure.

### Ultrasound-guided caudal block procedure

The US-CEB procedures were performed in a standardized manner to ensure consistency across all practitioners. All patients were placed in the prone position with a pillow under the pelvis. The skin over the sacral area was prepared with antiseptic solution, and sterile drapes were applied. Residents received hands-on instruction using a high-frequency linear array probe (6–15 MHz, HFL50x, Fujifilm SonoSite). Residents received hands-on instruction in ultrasound optimization: depth adjustment, gain settings, focus positioning, and color Doppler activation to identify vascular structures. Trainees practiced pre-scanning adjustments on standardized patients to achieve optimal visualization of sacral landmarks prior to live procedures.

**Figure 1:**
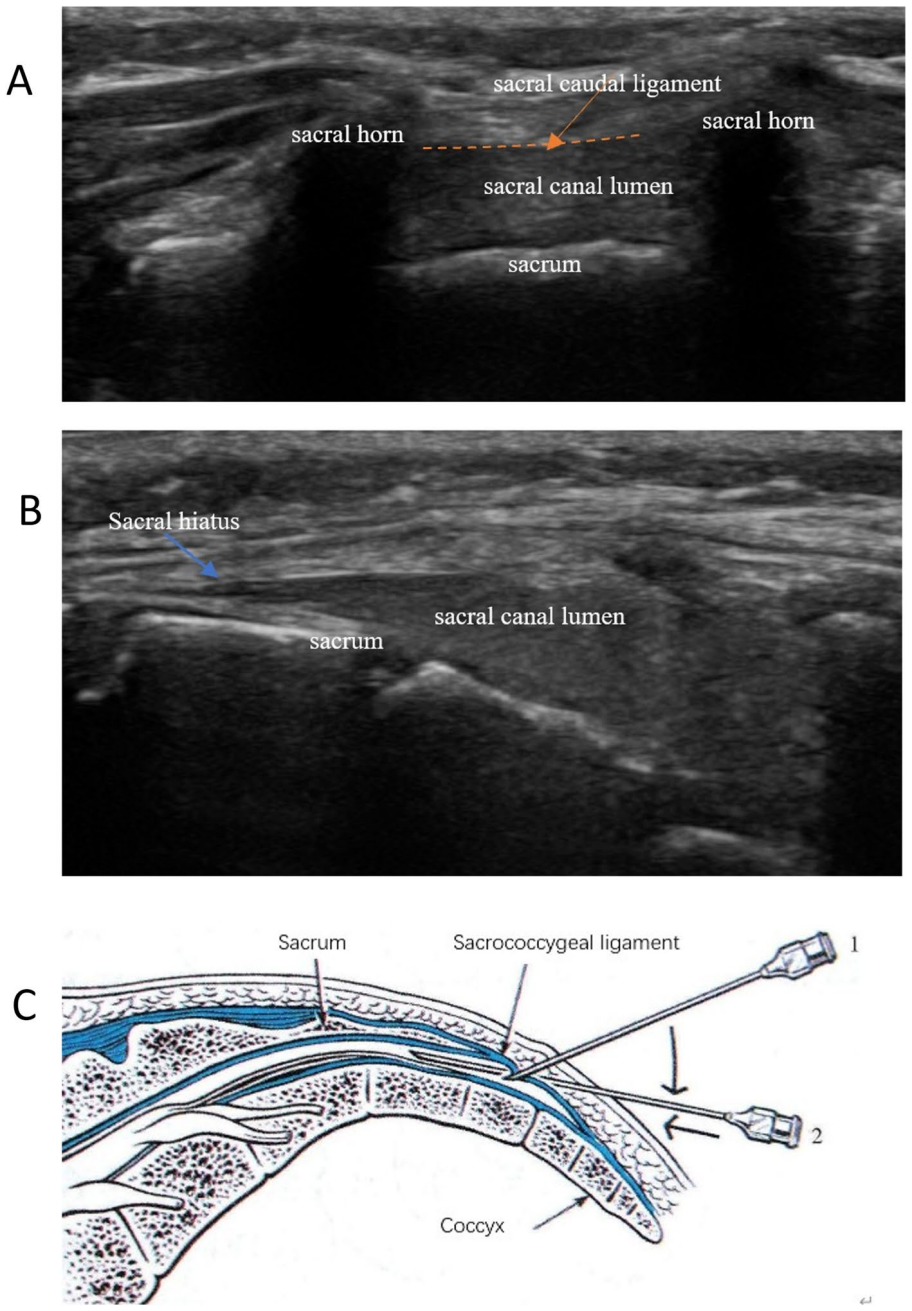
Typical ultrasound images of the sacral canal caudal block anatomy. **(A)** Short-axis views: frog’s face sign; **(B)** Long-axis views: staircase sign; **(C)** Ultrasound-guided intraplanar puncture technique for sacral canal block.

The ultrasonic probe was positioned in a transverse orientation to visualize the bilateral sacral horn, sacral caudal ligament, and sacrum, as well as to identify the sacral hiatus, which resembles a frog’s face (Figure 1A). Subsequently, the ultrasonic probe was rotated 90 degrees to obtain a longitudinal view of the sacral hiatus, sacral caudal ligament, and sacrum (Figure 1B). A 20-gauge, 80-mm needle was then inserted in-plane with the ultrasound probe, targeting the sacral hiatus through the sacrococcygeal ligament. Real-time ultrasound guidance was used to visualize the needle as it advanced through the sacrococcygeal ligament and into the epidural space, which allowed me to see the needle’s entire length, as well as loss of resistance technique using saline. The needle then was further gently advanced 0.5 cm (Figure 1C) to avoid spinal puncture. Once the needle tip was confirmed to be in the epidural space, a test dose of 2 mL of saline was injected to ensure proper placement. Following confirmation, 20 mL of 0.375% ropivacaine (Naropin; AstraZeneca, Sodertalje, Sweden) was administered slowly, with continuous ultrasound monitoring to ensure even distribution of the anesthetic. The efficacy of the block was evaluated by assessing sensory and motor blockade in the pertinent dermatomes.

### Quality control

The learning curve for US-CEB is influenced by several factors, including the operator’s prior experience with ultrasound imaging, the complexity of the patient’s anatomy, and the specific techniques employed during the procedure.

In a study investigating the learning curve, residents who had no prior experience in ultrasound. All residents are trained by the same senior teacher. To ensure a controlled and unbiased study, a preliminary assessment was conducted by a highly skilled physician. This expert utilized ultrasonography to identify sacral canal anatomical landmarks for each patient. This step was crucial for standardizing the patient cohort and ensuring uniformity in the study. Additionally, this process allowed for the exclusion of patients presenting with any anatomical variations that could potentially skew the results. By maintaining a consistent and rigorous selection criterion, the study aimed to minimize variability and enhance the reliability of the findings.

### Collected variables

The following variables were collected for each US-CEB procedure: (1) Patient demographics (age, gender, BMI, ASA status). (2) Success rate of the block (defined as effective anesthesia for surgery). (3) The landmarks Identification time: defined as the time from the contact of the ultrasound probe with the patient to correctly identify short-axial the frog’s face sign and the long-axial sacral lumen. (4) US-CEB procedure time: defined as the time from the contact of the needle with the patient to the sacrococcygeal ligament and into the epidural space. (5) Procedure self-confidence was scored by the operating resident immediately after each block using a 10-point scale (1: very low confidence; 10: very confident). While self-assessment introduces subjectivity, this approach is validated in skill-acquisition studies and reflects the operator’s perceived readiness for independent practice (11, 12). Complications (dural puncture, intravascular injection, incomplete block). All were recorded with stopwatch by teacher in the same area. The learning curve was plotted with the average time at the same number of 10 novice anesthesiology residents.

### Statistical analysis

Descriptive statistics were used to summarize patient demographics and procedural variables. Continuous variables were expressed as mean ± standard deviation (SD), and categorical variables were expressed as frequencies and percentages. Based on the pre-experimental data [*α* = 0.05, *β* = 0.2, effect size d = 0.8 (13)], calculated by G*Power 3.1, each group requires at least 9 operations. To account for inter-operator variability and ensure robust phase identification in CUSUM analysis, we expanded the sample size to 30 procedures per resident-a common threshold in procedural learning curve studies (consistent with findings by Barrington (7), who used 30 cases to stabilize learning curve estimates in ultrasound-guided brachial plexus blocks). All statistical analyzes were performed using IBM SPSS Statistics 29 (IBM Corp., Armonk, NY, United States). To evaluate the learning curve for US-CEB, the Cumulative Sum (CUSUM) analysis was employed.

### CUSUM analysis

The CUSUM analysis is a robust method for assessing the acquisition of procedural skills, allowing for continuous monitoring of performance over time. In clinical practice, CUSUM monitors outcomes like complications or success rates, with the capacity to adjust for patient risk. It offers early warnings of performance changes, helping to maintain quality by addressing issues promptly. This method allows for proactive quality control, can be risk-adjusted for patient variability, and aims to maintain service quality by detecting issues early. To assess the potential for overfitting and evaluate the generalizability of the polynomial models, we performed leave-one-out cross-validation (LOOCV). The cross-validated *R*^2^ (*R*^2^_cv) values are reported alongside the standard *R*^2^ to provide a more robust estimate of model performance. The CUSUM method was used to analyze the learning curve. First, the patients were placed in chronological order from the earliest to the latest date of surgery. Data for each patient in the series were plotted on a chart from left to right on the horizontal axis. The CUSUM of the operation time (CUSUM CUSUMOT) was defined as follows:


$$\text{CUSU}M_{\mathrm{OT}}=\sum_{i=1}^{n}(x_{i}-\mu )$$

where x_i_ is the patient’s individual operation time and *μ* is the mean overall operation time of all patients. Thus, CUSUM_OT_ of the first patient was the difference between the operation time for the first patient and the mean operation time for all patients. This recursive process was continued until CUSUM CUSUM_OT_ of the last patient was calculated as zero. The learning curve with respect to the landmarks Identification time and the US-CEB operation time was represented intuitively and determined by plotting the outcomes on the CUSUM curve. The inflection point (proficiency threshold) of the CUSUM curve was identified via visual inspection of the slope transition, supported by the Chow test to confirmed a significant difference in regression coefficients before and after the proposed inflection points (*p* < 0.01 for all three metrics: landmark identification, procedural time, and self-confidence). Risk-adjusted CUSUM parameters were defined as follows: Acceptable failure rate p_0_ = 0.20 (based on novice performance benchmarks); Unacceptable failure rate p_1_ = 0.40 (twice p_0_); Type I error *α* = 0.05, Type II error *β* = 0.10; Reference value k = 0.5, Decision limit h = 4.77. These yield an in-control Average Run Length (ARL₀) of 200 procedures when the true failure rate is 20%, ensuring low false-alarm risk. The CUSUM values were plotted against the number of procedures performed by each anesthesiologist. The plot provides a graphical representation of the learning curve, with the x-axis representing the number of procedures and the y-axis representing the CUSUM value.

### Phases of learning curve

The learning curve was divided into two phases based on the CUSUM plot: Initial Phase: Characterized by high variability in procedure time and frequent deviations from the proficiency. Proficiency Phase: Marked by a steady decline in the CUSUM value, indicating increasing proficiency and minimal deviations from the target.

## Results

This study involved a cohort of 10 anesthesiology residents who held comparable bachelor’s degrees in anesthesiology and underwent identical foundational training. Pre-study assessments confirmed uniform baseline US-CEB inexperience. While individual aptitude may vary, our standardized curriculum and supervised practice mitigated confounding effects of self-learning variability. The study was conducted over a period of 4 months, during which each anesthesiologist performed a minimum of 30 US-CEB procedures. The patient population included in this study consisted of 300 individuals undergoing perineal and sacrococcygeal surgeries. The demographic characteristics of the patients were as follows: the mean age was 43.6 years (range 24–65 years), with a gender distribution of 65% male and 35% female. The inclusion criteria for patients were based on the type of surgery and the absence of contraindications for caudal block anesthesia. There were no significant differences in sex, age, height, weight or BMI of the patients being procedure by participants. The duration of the first and second inter-training sessions were 3.6 (1–7) days.

The median landmarks Identification time of all participants was 49.5 s (22–259 s). The median US-CEB procedure time of all participants was 146.6 s (65–425 s). Additionally, two cases of transient local anesthetic intoxication, presenting as dizziness, were observed and resolved without the need for intervention.

### Learning curve analysis and phase

**Figure 2:**
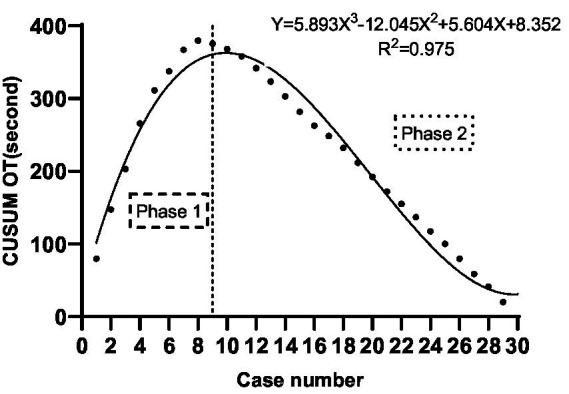
CUSUMOT of scanning landmarks identification versus number of patients.

The CUSUM CUSUM_OT_ learning curve of scanning landmarks Identification time was best modeled as a third-order polynomial (parabola) with the following equation: CUSUM CUSUM_OT_ (in seconds) = 5.893 × patient number^3^−12.045 × patient number^2^ + 5.604 × patient number+8.352. This had a high *R*^2^ value of 0.975. According to the change in the slope shown in Figure 2, we divided the CUSUM learning curve into two unique phases: phase 1 (the initial 9 patients), phase 2 (the final 21 patients).

**Figure 3:**
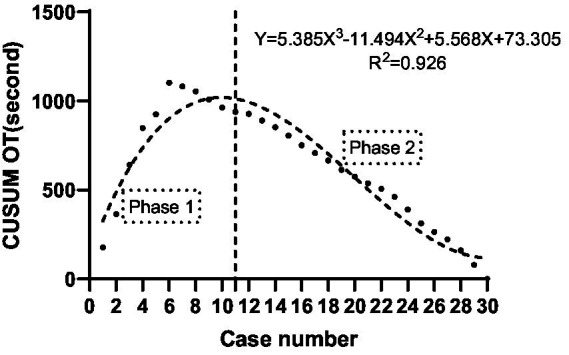
CUSUM_OT_ of US-CEB procedure time versus number of patients.

The CUSUM CUSUM_OT_ learning curve of procedure time was best modeled as a third-order polynomial (parabola) with the following equation: CUSUM CUSUM_OT_ (in seconds) = 5.568 × patient number^3^−11.494 × patient number^2^ + 5.568 × patient number + 73.305. This had a high *R*^2^ value of 0.926. According to the change in the slope shown in Figure 3, we divided the CUSUM learning curve into two unique phases: phase 1 (the initial 11 patients), phase 2 (the final 19 patients).

**Figure 4:**
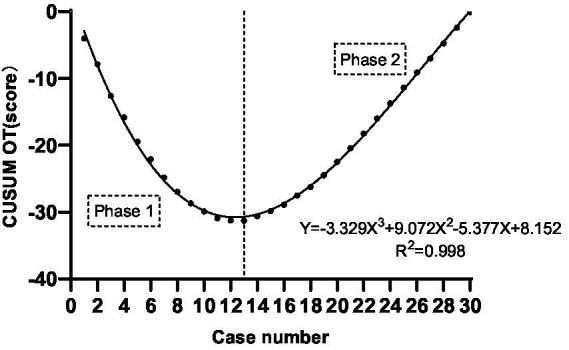
CUSUM_OT_ of self-confidence score operating versus number of patients.

The CUSUM CUSUM_OT_ learning curve of self-confidence score operating was best modeled as a third-order polynomial (parabola) with the following equation: CUSUM CUSUM_OT_ (in seconds) = −3.329 × patient number^3^ + 9.072 × patient number^2^−5.377 × patient number+8.152. This had a high *R*^2^ value of 0.998. According to the change in the slope shown in Figure 4, we divided the CUSUM learning curve into two unique phases: phase 1 (the initial 13 patients), phase 2 (the final 17 patients).

Table 1 compares the preoperative parameters among the two phases of US-CEB procedure learning curve. No significant differences were observed in any of the preoperative data (age, sex, weight, height, BMI). Thus, the patients in all two phases were considered comparable with respect to the preoperative data.

**Table 1:** Preoperative parameters among the learning curve phases.

	Phase 1 (*n* = 110)	Phase 2 (*n* = 190)	*p* value
Age (years)	43.32 (9.15)	44.37 (8.76)	0.14
Gender (%, male)	59.41	58.29	0.83
Height (cm)	162.94 (7.08)	163.63 (7.83)	0.70
Weight (kg)	60.27 (9.33)	62.43 (8.41)	0.32
BMI (kg/m^2^)	22.74 (3.50)	23.31 (2.79)	0.46

kg, kilogram; cm, centimeter; BMI, body mass index; References.

Table 2 compares residents’ performance among two learning curve phases for scanning landmarks identification, US-CEB procedure, and self-confidence score operating, which is significantly improved, with statistical significance. The scanning landmarks identification time decreased significantly between phase 1 (84 s; range, 38–122 s) and phase 2 (24.38 s; range, 21–25 s; *p* < 0.001) in the scanning landmarks identification learning curve. US-CEB procedure time decreased significantly between phase 1 (242.9 s; range, 102–425 s) and phase 2 (98.45 s; range, 65–135 s; *p* < 0.001) in the US-CEB procedure learning curve. The self-confidence score increased significantly between phase 1 (5.12; range, 3.5–7.5) and phase 2 (9.36; range, 7.5–9.8; *p* < 0.05) in the self-confidence score learning curve.

**Table 2:** Two learning curve phases for scanning landmarks identification, US-CEB procedure, and self-confidence score operating.

	Phase 1	Phase 2	*p* value
Scanning landmarks Identification time (sec)	84 (27.681)	24.38 (3.598)	<0.001
US-CEB procedure time (sec)	242.9 (123)	98.45 (18.523)	<0.001
Self-confidence score operating	5.12 (1.477)	9.36 (0.588)	<0.05

US-CEB, ultrasound-guided continuous epidural block; sec, second.

## Discussion

The primary aim of this study was to evaluate the learning curve of anesthesiologists performing ultrasound-guided caudal epidural blocks (US-CEB) using CUSUM (Cumulative Sum Control Chart) analysis.

The CUSUM analysis is a robust method for assessing the acquisition of procedural skills, allowing for continuous monitoring of performance over time. Our study demonstrated that novice residents, with no prior experience in US-CEB, were able to achieve a significant improvement in their procedural skills over a relatively short period. The median time for landmark identification and procedure execution decreased substantially from phase 1 to phase 2, indicating a steep learning curve. This is consistent with previous studies that have reported a rapid improvement in ultrasound-guided regional anesthesia skills with deliberate practice (7, 14). In this study, the learning curve for landmark identification plateaued after approximately 9 procedures, and for the US-CEB procedure itself after approximately 11 procedures. These results align with previous studies that have reported a similar number of cases required to achieve proficiency in ultrasound-guided regional anesthesia techniques. For instance, a study by Kollmann A et al. (13) showed that a plateau in performance was reached after performing around 12 ultrasound-guided femoral nerve blocks.

The use of the Cumulative Summation Chart for Outcomes by Time (CUSUM) learning curve analysis allowed us to model the residents’ progress as a third-order polynomial, revealing two distinct phases of learning. This method is more sensitive than traditional linear regression in detecting changes in performance over time (15). The high *R*^2^ values (0.975 for landmark identification, 0.926 for procedure time, and 0.998 for self-confidence score) indicate a strong fit of the model to the data, validating the use of polynomial functions in analyzing learning curves. The parabolic shape of the curves indicates an initial rapid improvement followed by a plateau, which is typical of learning curves in procedural skills (16).

Dividing the learning curve into two phases, as evidenced by changes in the slope of the CUSUM curves, allows for a more nuanced understanding of the learning process (17). During the initial phase, residents experienced a steep decline in the time taken for both landmark identification and procedure execution. This rapid improvement can be attributed to the residents’ initial exposure to the technique and their focus on mastering basic skills such as probe handling, image acquisition, and needle guidance. The significant reduction in procedure time from phase 1 to phase 2 is indicative of the residents’ ability to integrate these skills into a coherent procedural workflow. This phase is critical for establishing foundational skills and confidence. In the second phase, the learning curve plateaus, suggesting that residents have reached a level of proficiency where further improvements are incremental. This phase is characterized by the refinement of motor skills and the development of situational awareness. The residents’ increased self-confidence, as evidenced by the significant rise in self-confidence scores, is a key indicator of their growing autonomy and readiness for independent practice. This division is useful in educational programs as it allows for targeted reinforcement of skills in the initial phase and consolidation of knowledge in the final phase (17).

The self-confidence scores of the residents increased with the accumulation of the number of operations (18). The self-confidence score learning curve suggests that the increase in self-confidence among trainees is closely related to their procedural experience. This is an important finding, as self-confidence is a critical factor in the transition from novice to expert. The fact that self-confidence continues to increase even after the plateau in procedural time suggests that ongoing experience and feedback contribute to a deeper understanding and mastery of the technique. Previous studies have similarly highlighted the importance of self-assessed confidence in skill acquisition (18).

The lack of significant differences in preoperative parameters (age, sex, weight, height, BMI) between the two phases of the US-CEB procedure learning curve indicates that the progression of learning was not influenced by patient characteristics. This finding is crucial as it suggests that the learning curve is a reflection of the trainee’s skill development rather than variations in the patient population. This consistency in patient demographics supports the validity of the learning curve analysis.

The absence of serious complications and the low incidence of transient local anesthetic intoxication in our study are encouraging. This suggests that the learning process did not compromise patient safety, which is a critical consideration in any training program. The findings underscore the importance of structured training and supervision in ensuring that procedural skills are acquired without compromising patient outcomes.

Our findings offer concrete data to inform the structure of regional anesthesia training. The learning phases we identified suggest that trainees require a minimum of 9 to 11 supervised procedures to achieve basic technical proficiency in US-CEB. Therefore, we recommend that training curricula mandate at least this number of proctored practices before allowing trainees to perform the procedure with minimal supervision. Furthermore, since self-confidence plateaued later than technical skill, educators should be aware that a trainee’s technical competence may precede their confidence. Incorporating structured confidence assessments alongside technical metrics can provide a more holistic view of a trainee’s readiness for independent practice.

### Limitation

Despite the considerable insights provided, our study on the learning curve of US-CEB has its inherent limitations. The relatively small sample size of residents and the single-center design limit the generalizability of our findings. Future studies with larger cohorts and multicenter designs are needed to validate our results. Additionally, long-term follow-up studies are required to assess the durability of the skills acquired during the learning phase. Additionally, exploring the impact of other factors such as trainee motivation, prior experience in anesthesia, and the role of mentorship in the learning process would enrich our understanding of the factors influencing skill acquisition in regional anesthesia.

In conclusion, the study demonstrates a relatively short learning curve for US-CEB, emphasizing the feasibility of proficiency achievement through focused training. The structured training program during the initial phase and the deliberate practice program in the subsequent stages provide pivotal guidance for residents learning the intricacies of ultrasound-guided sacral canal block procedures. As an operator’s skill set expands through learning and practice, their confidence in their abilities also grows, creating a positive cycle that supports ongoing skill development and mastery.

## Data Availability

The raw data supporting the conclusions of this article will be made available by the authors, without undue reservation.
